# Bioproduction and applications of aldobionic acids with a focus on maltobionic and cellobionic acid

**DOI:** 10.1007/s00449-023-02872-7

**Published:** 2023-04-14

**Authors:** Emmeran Bieringer, Uxía García Vázquez, Luisa Klein, Núria Moretó Bravo, Matthias Tobler, Dirk Weuster-Botz

**Affiliations:** 1 TUM School of Engineering and Design, Department of Energy and Process Engineering, Chair of Biochemical Engineering, Technical University of Munich, Boltzmannstr. 15, 85748 Garching, Germany; 2 TUM School of Engineering and Design, Technical University of Munich, Boltzmannstraße 15, 85748 Garching, Germany; 3 TUM School of Natural Sciences, Technical University of Munich, Lichtenbergstraße 4, 85748 Garching, Germany; 4 TUM Campus Straubing for Biotechnology and Sustainability, Technical University of Munich, Petersgasse 5, 94315 Straubing, Germany

**Keywords:** Cellobionic acid, Maltobionic acid, Plant-based supplements, Whole-cell biocatalysis, Enzymatic biocatalysis

## Abstract

Aldobionic acids are sugar acids which consist of a disaccharide with an anomeric acid group. The most famous is lactobionic acid (LBA). LBA is used in many applications such as food and beverages, pharmaceuticals and medicine, cosmetics or chemical processes. During the last decade, all these industries are observing a shift of consumer preferences towards plant-based options. Thus, the biotechnological industry is trying to replace the animal-derived LBA. Maltobionic acid (MBA) and cellobionic acid (CBA) are two stereoisomers of LBA which have emerged as vegan alternatives. However, MBA and CBA face different obstacles related to their industrial production. While traditionally used electrochemical or chemical catalysis often rely on cost intensive and/or hazardous catalysts, novel production methods with microorganisms are still poorly studied. In the first part, this paper discusses both alternatives in terms of their characteristics and applications. In the second part, it reviews the long-studied chemical production and the novel bioproduction methods, which are based on enzymatic and microbial systems. This review concludes with a discussion of future work needed to bring their production to the industrial scale.

## Introduction

Sugar acids are organic acids derived from the direct oxidation of mono- or oligosaccharides such as gluconic acid, and lactobionic acid (4-O-β-galactopyranosyl-D-gluconic acid; LBA), respectively [1]. Acids of bioses are called bionic while those of aldoses aldonic acids. LBA is a combination of both: an aldobionic acid. It has several properties which make it especially interesting for the cosmetic, pharmaceutical, food, and chemical industry [2]. Other aldobionic acids are maltobionic acid (4-O-α-D-glucopyranosyl-D-gluconic acid, MBA), obtained from maltose oxidation, and cellobionic acid (4-O-β-D-glucopyranosyl-D-gluconic acid; CBA) as product of cellobiose oxidation [2]. Both of them are stereoisomers of LBA, thus they have similar physicochemical characteristics [1, 3, 4]. These similarities make MBA and CBA potential suitable replacements.

**Fig. 1:**
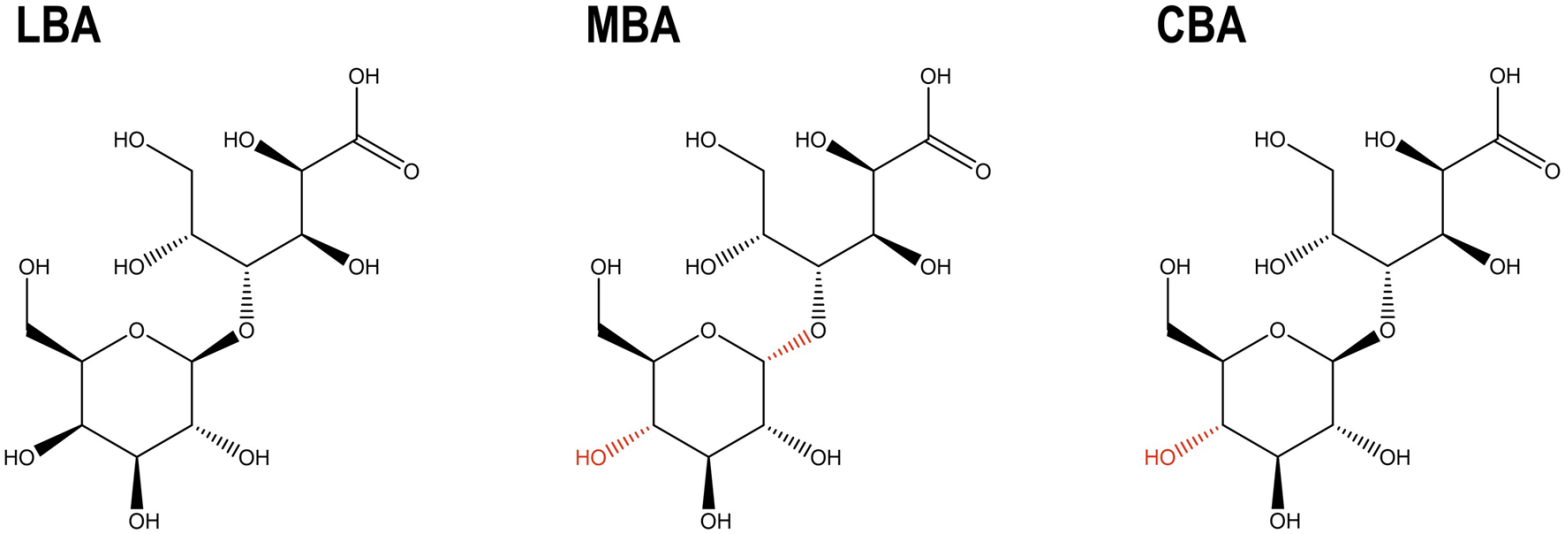
The structure of lactobionic acid (LBA) and its stereoisomers maltobionic (MBA) and cellobionic acid (CBA) [5–7]. The differences of MBA and CBA compared to LBA are highlighted in red (colour figure online)

All of these aldobionic acids (Fig. 1) have a 1,4-glycosidic bond, the same molecular weight of 358.30 g mol^−1^ and a pK_a_ of 3.86 [5, 6].

The acids have multiple chiral hydroxyl groups. Aldobionic acids are hydrophilic and almost insoluble in organic solvents. Also, due to the functional groups and corresponding hydrogen bonds, moisturizing and moisture absorbing properties were observed [8, 9]. Furthermore, MBA can easily bind and solubilize minerals [10], increasing especially the absorption of calcium (Ca^2+^), magnesium (Mg^2+^) and iron (Fe^3+^) [11, 12]. As these molecule is a combination of a saccharide and an acid, MBA have one fifth of the acidity of citric acid [10] and show slight sweetness [13]. A study on rats and humans shows that MBA is almost indigestible and resistant to fermentation by gut microbiotica [11].

Summarized, MBA and CBA have several similar physicochemical properties in common with LBA: All are biocompatible, biodegradable but partially indigestible, antioxidative and antimicrobial, non-toxic, highly water soluble, moisturizing [1, 8, 9, 14, 15], metal chelating, mildly sour [11] and slightly sweet [13]. As a result, MBA and CBA can be applied in the same sectors as LBA. The latter is used in pharmaceutics, cosmetics, food and beverages as well as chemical industry [2, 14]. In various applications, the plant-derived MBA and CBA can be considered as substitutes for animal-derived ingredients like LBA. During the next years, their demand may rise constantly due to the fast expansion of the plant-based consumer good market. For instance, the market value of vegan personal care products is estimated to rise from $15.87 billion in 2021 to $24.79 billion in 2028 [16]. Regarding food and beverages products, both sugar acids could achieve even more importance because the global plant-based food market (plant-based dairy, plant-based meat and others) is expected to grow from $29.4 billion in 2020 to $161.9 billion in 2030 [17]. Consequently, the forecasted increase in the demand of MBA and CBA leads to the need of efficient production methods.

Traditionally, these types of acids have been produced via electrochemical or chemical oxidation methods. However, from an industrial perspective, the scale-up of these methods is affected by the use of harmful and/or expensive catalysts and the generation of unwanted side-products. Recent research has been directed towards the development of new bio-production methods classified according to the biocatalysts used, microbial *vs.* cell-free systems [18]. The main difference between microbial, also known as whole-cell, and cell-free systems is the ability of regeneration: The biocatalyst in whole-cell fermentation is continuously regenerated, whereas cell-free biocatalysts must use different strategies to prolong their lifetime [19], like thermostable enzymes, immobilization, and enzyme engineering [20]. A summary of the differences between whole-cell and cell-free systems is compiled in Table 1.

**Table 1:** Bio-production of aldobionic acids classified according to the biocatalysts used, microbial and cell-free systems; compilation derived from [18]

Metric	Cell-free system	Whole-cell system
Pathway engineering	+ Use of all of substrate’s chemical energy due to synthetic pathways − higher yield possible + Chimeric pathway approaches: Simultaneous use of enzymes from different hosts, no restriction by difficult expression or essential post-translational modifications	+ Ability to harness the power of evolution (continuous culture, directed evolution, etc.) − Opposition of engineering objective and microbe’s aim of growing; compromise necessary − Difficult to predict the effects of modified pathways in vivo
Reaction control	+ Easier control of reaction conditions: Temperature, pH, substrate concentration, etc	+ Replacement of labile enzymes and cofactors by self-replicating biocatalysts
Cell wall	+ No transport limitations regarding substrate or product	+ Membrane proteins available − Energy dependant transport of the substrate and product across cell membrane
Maturity	+ Simpler system; nearly complete basic knowledge of the system − Recently established	+ Many established conversions + Extensive practical knowledge
Toxin and solvent effect	+ Higher tolerance towards longer-chained alcohols/solvents	− No or suppressed growth or product production by microbe
Cost	+ No large-scale lysis step required in downstream processing − Low catalyst costs required; need to use enzymes with high turnover number, cheap production and purification methods, and low amounts of thermolabile cofactors and expensive substrates	+ No cell lysis step required, when using membrane-bound enzymes for conversion or product is secreted into the medium + Wide range of complicated biological precursors (amino acids, polysaccharides, etc.) available for low cost
Scale-up	+ Similar system behaviour at lab and larger scales	− Risk of failure due to phage contamination − Management of strain evolution

Cell-free systems can also be divided into cell extracts or purified enzymes. Purified enzyme systems include the over-production of the enzymes and subsequent purification from the native organism. By contrast, in a cell extract method, enzymes are over-expressed and cell lysate is used as the catalyst. Using cell extracts or purified enzymes instead of microbial fermentation, different advantages can be achieved, such as the straightforward control of the reaction conditions specific for the enzymatic reaction, the reduction of toxicity effects of reactants on cell metabolism or that there is no large-scale cell lysis required in subsequent downstream processing [18]. Although an enzymatic approach is more selective and efficient, with whole-cells, no disruption and purification of enzymes is necessary, which saves resources and process time [21], thereby reducing production costs for the biocatalysts considerably. The stability of the enzymes is also higher within cells and the cofactor regeneration is better managed but in contrast, a cell-free system does not have to deal with mass transport limitations across the cell membrane. Without a whole metabolism, less side reactions and no contaminations by cellular products occur so that the costs for downstream processing may be reduced [21].

Nevertheless, MBA and CBA have a significant drawback in the sense that the biocatalysts reported so far are not very efficient. Therefore, there is an urgent need to accelerate the screening and exploration of new strains with improved biocatalytic activities. This review offers an analysis of the chemical and especially bio-production methods for MBA and CBA and gives a detailed overview of possible fields of applications. Furthermore, a section of feasible downstream methods for the recovery of MBA and CBA is presented.

## Applications

**Fig. 2:**
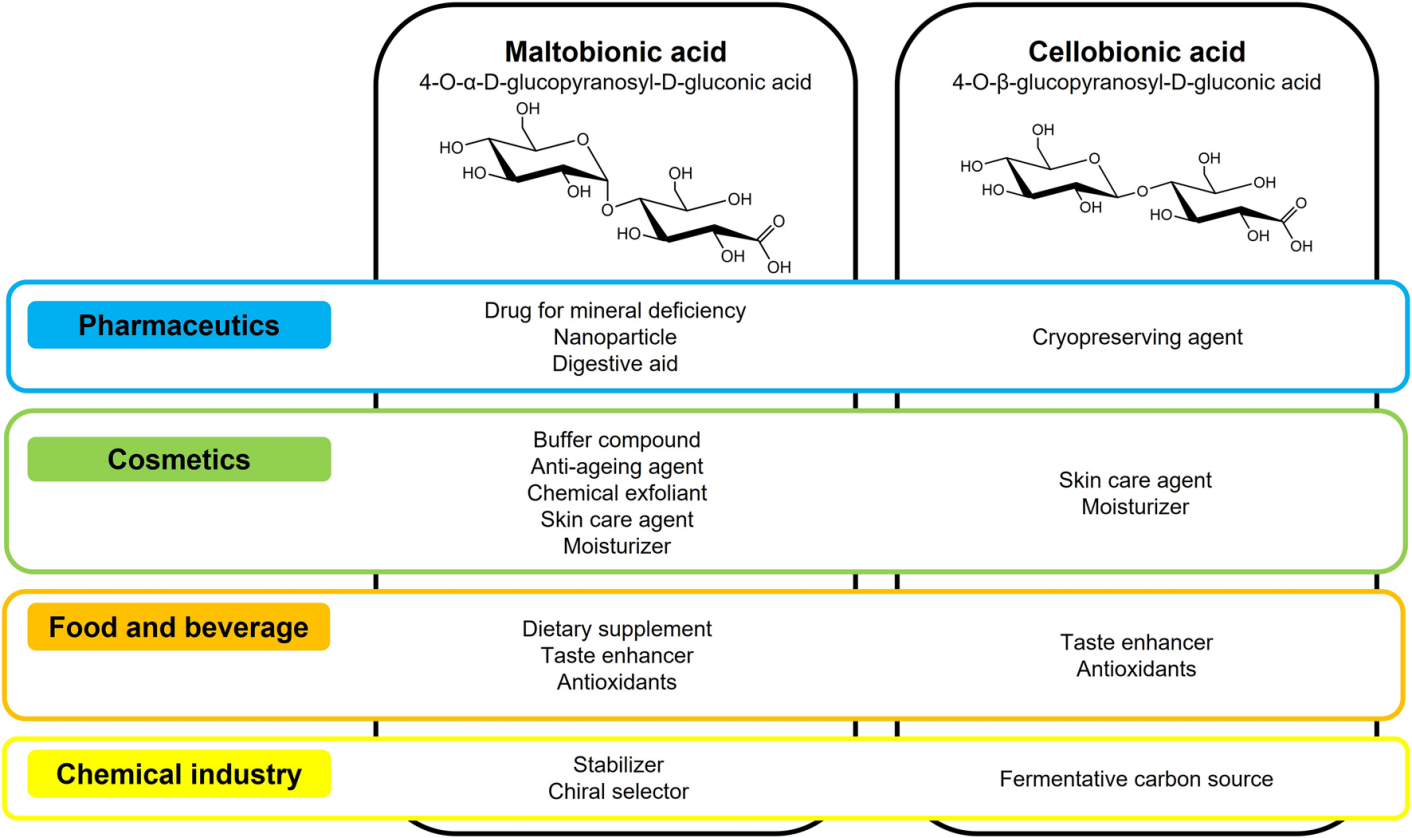
Summary of possible applications of MBA and CBA in the domain of pharmaceutics, cosmetics, food and beverages as well as chemical industry (own compilation, with chemical structures derived from [5, 6])

As previously mentioned, the physicochemical properties of LBA, MBA and CBA are similar, leading to the fact that MBA and CBA can be applied in the same domains as LBA. Possible applications of MBA and CBA are displayed in Fig. 2.

### Application in pharmaceutics

Most of the indicated pharmaceutical applications in Fig. 2 refer to MBA since CBA has not yet been investigated deeply. In pharmaceutics, MBA is of interest because it improves the solubility of cations such as Ca^2+^, Fe^3+^ and Mg^2+^. As MBA is not absorbed in the intestine, the minerals remain dissolved and are available for absorption. Therefore, MBA leads to a more efficient mineral uptake [11, 12, 22]. This effect is beneficial to promote bone mineralisation. Suehiro et al. measured a rise in Ca^2+^ concentration in the femurs of rats by feeding the animals with maltobionic acid calcium salt (MBA-Ca) [11]. Higher femoral Ca^2+^ levels were achieved if at least 50% of the total Ca source was MBA-Ca, suggesting that MBA-Ca provides a higher mineralization efficiency compared to other Ca sources like CaCO_3_. Similar results have been found in a human trial with 34 healthy, postmenopausal women. Each day, they received either a corn syrup solid containing MBA-Ca or a placebo. After 24 weeks, the bone mineral density (BMD), the bone area and the bone mineral content of 19 remaining participants improved [23].

The effect of MBA on bone metabolism was investigated in another study with a similar set-up including 26 women, showing that MBA has a positive impact on bone metabolism by averting bone resorption [24]. It is thought that this effect is caused by the increased calcitonin, as this hormone is responsible for restraining bone resorption [25]. Highlighting the benefits of MBA on bone health suggest that its ingestion may prevent osteoporosis, a disease accompanied by a low BMD and an extended risk of fractures [26, 27]. Osteoporosis is caused by bone metabolic imbalance and is a major global health problem which is more prevalent in people more than 50 years old [13, 15]. In times of demographical ageing, the importance of MBA should be thus emphasised. In general, osteoporosis is pharmacologically treated by bisphosphonates, monoclonal antibodies such as Denosumab, hormonal therapies with estrogenic agonists/antagonists, calcitonin or parathyroid hormone analogues [28]. To reduce possible side effects of this common medication, additional ingestion or partial substitution with MBA would be advantageous and should be considered in future osteoporosis therapy [12].

Another important health issue is anaemia, which concerns approximately 29.9% of women between the ages of 15 and 49 years globally as of 2019 [29]. According to the WHO, one of the leading causes of anaemia is iron deficiency [30]. Suehiro et al. demonstrated recently that rats fed with MBA-Ca recover more easily from latent iron deficiency or iron-induced anaemia, revealing MBA-Ca as a possible candidate to cure anaemia [22]. A reason for this finding could be that MBA enhances the solubility of iron and thus improves the iron uptake after Ca has been released and absorbed in the small intestine [22]. Likewise, a regular administration of MBA lowers the constipation symptoms by increasing the stool frequency and softness as well as bowel movements and thus contributes to human well-being [13].

Even if MBA seems to be health promoting, it has to be taken to account that the number of participants in all the presented human trials was small [13, 23, 24], or that there is no evidence for health benefits in humans yet [11, 22]. It is necessary to point out that all the mentioned publications involved similar authors. Additionally, there was a conflict of interest at a publishing date because two of the authors were working for Sun-ei Sucrochemical Co. Ltd., a company producing a syrup and a candy containing MBA [31].

Furthermore, MBA is a key compound in a therapeutic nanoparticle devised for treating brain cancer [32]. The core elements of this concept are small multiple telodendrimer micelles carrying the drugs and functionalized with MBA and 4-carboxyphenylboronic acid (4-CPBA) in a molar ratio of 9:1. By cross-linking MBA and 4-CPBA, the micelles are connected, resulting in a polymeric nanoparticle. Apart from nanoparticle formation, MBA is essential to make the nanoparticle pass the blood–brain barrier/blood–brain tumour barrier [32]. This stems from the fact that MBA, as a glucose derivative, is recognized on the surface of the nanoparticle by the facilitative glucose transporter 1 (GLUT1) which allows its transcytosis [32, 33]. Beyond the barrier the low pH in tumour tissue causes the release of the micelles, which can then enter tumour cells via 4-CPBA [32]. The developed nanoparticle lies under patent since June 2021 [34].

Even though CBA could be potentially employed in pharmaceutical industry, it has not yet received much attention. Only one patent could be found involving the use of CBA as one compound in liquid cryopreservation of stem cells [35]. Showing similar physicochemical properties, it is highly probable that MBA and CBA can be swapped reciprocally.

Another important aspect is that the risks accompanying the intake of MBA and CBA have not yet been intensively investigated. Indeed, Fukami et al. demonstrated that MBA has no negative impacts on human health, if 8 g of MBA are ingested daily for 24 weeks [23]. However, further risk assessments are still necessary to prove the health safety of both aldobionic acids at short and long-term perspectives.

### Applications in cosmetics

Based on the available references, the majority of the herein presented cosmetic applications are only published in patents.

In the cosmetic industry, MBA and CBA are employed as additive to promote the functionality and stability of the target product. For instance, MBA serves as one of the buffer compounds in a skin whitening formulation [36]. MBA was also proposed as one possible conserving agent for cosmetic or pharmaceutical products as well as for washing or cleaning agents which are susceptible to acids [37]. Evaluating the minimum inhibitory concentration of MBA on different bacterial species, 4–12 mg ml^−1^ of MBA were sufficient to inhibit growth of different bacteria [37]. The antimicrobial effect of MBA is not a result of acidity since the pH only decreased from pH 7.6 to pH 6.5–pH 5.5 during the test. Thus, MBA is said to suppress microbes because it destabilizes the bacterial cell wall, complexes iron and binds water, leading to a decrease of their bioavailability. To avoid microbial spoiling, cosmetic products with pH 4–pH 7 could be supplemented with 0.2–4.0% (w/v) of MBA, depending on the product’s total weight [37]. Hair products exploit the chelating properties of acids including sugar acids. Metal salts of MBA or CBA with iron or zinc exercise hair strengthening effects, because the aldobionic acid stabilizes the metal ion. The latter enhances hair’s thermal and mechanical resistance by interacting with keratin [38]. Blended in hair dye, those metal salts help additionally to maintain the hair colour [39].

Similar to LBA, the chelating and thus antioxidant properties of both sugar acids may protect against skin aging [40, 41]. This tissue degradation is considered to be caused by reactive oxygen species (ROS) [42, 43]. ROS are generated via intracellular oxidative processes which can be induced by UV exposure, a predominating extrinsic factor of skin aging [44]. Releasing iron ions from metalloproteins, UV radiation initiates their oxidation [45]. In combination with H_2_O_2_, hydroxide ions as well as hydroxyl radicals with high reactivity are formed [40, 44].

Lipid peroxidation is characterized by a loss in membrane integrity as well as cellular and metabolic functions [46, 47]. Thus, complexing iron ions would help to reduce skin aging. As a result, both sugar acids are promising cosmetic anti-aging agents. Notably, a novel face mask employed MBA as active cosmetic ingredient to prevent dermal degradation [48].

Being hygroscopic, MBA and CBA show a high-water uptake. The resulting gel matrix would be advantageous in skin care products treating inflammation and wounds [49]. Their moisturising effect may also be useful to overcome or prevent skin dryness [50].

Furthermore, exfoliant peelings and other similar products already employ MBA as one of the ingredients to improve skin clarity [48, 49]. In general, aldobionic acids are said to be beneficial active ingredients in peelings because they allow epidermal cells to renew faster and support skin regeneration [9, 51]. Additionally, polyhydroxy acids like LBA, MBA and CBA can be used to cure skin diseases like rosacea [52, 53]. It is also very probable that both sugar acids could serve to cure dermatological diseases, as atopic dermatitis or acne, like their close relative LBA [54, 55].

Overall, aldobionic acids are gentle to the skin and prevent skin irritation [56]. MBA and CBA could similarly be suitable for sensitive skin or for regenerative care products after harsh treatments like epilation or microdermabrasion [9, 56, 57].

### Applications in food and beverages

In recent years, the plant-based diets as “Veganism” and “Vegetarianism” have been steadily gaining popularity [58]. People are switching to these types of diets for health, ethical or environmental reasons. However, plant-based alimentation can come along with side effects like mineral deficiency and the risk of a lower BMD [59]. To avoid those disadvantages, the metal salts of MBA and CBA could become one of the key components since they increase the bioavailability of minerals, as mentioned before [11, 12, 22]. In particular, MBA-Ca has been shown to serve as functional food because it improves BMD as well as liver function [24, 55]. Being non-digestible and poorly used in microbial fermentation, MBA results in low energy content of only 0.46 kcal g^−1^ [60]. Thus, it could be used in diet food.

In terms of functional food, CBA is not yet employed but in contrast it is used as food additive. MBA and CBA serve both as an agent to ameliorate the taste of convenience food and beverages [10]. Already since 1974, the taste of solid–liquid and solid food is enhanced by adding a mixture of MBA and monosodium glutamate or MBA and maltose. Thanks to the weak own taste of MBA, it can be added in high quantities without being detected [61]. Moreover, there exist several pending patents that uses these sugar acids as food additive. For instance, they proposed supplementing dairy products with MBA or CBA to maintain and enhance the flavour of milk during processing [62]. In another patent, they are added to emphasise the saltiness of food products and allow to maintain or reduce the overall salt content [63]. MBA and CBA are additionally suggested to minimize the bitter taste of alcoholic drinks without increasing their sweetness [64]. Likewise, they can cover the bitterness of ingredients such as amino acids or amino acid metabolites [65]. In a pending patent, at least 0.4% (w/v) of MBA or CBA are added to drinks containing bean proteins because conventional agents are not able to overcome the specific unfavoured smell or taste of beans [66]. Taken together, MBA and CBA may not only be used to improve the flavour of food and beverages, but its supplementation could suppress or mask undesired tastes.

Moreover, the presented aldobionic acids could ensure the quality and stability of processed food or drinks, as indicated by two other patents: If eggs are one of the main ingredients in a drink or food, darkening may occur during the heating steps while processing. To avert this, MBA or CBA can be added since they complex iron which is involved in the unwanted colour change. The blending improves the texture of egg food too [67]. Another example is protein coagulation induced by MBA or CBA which leads to a more favourable consistency of the product without harming the resulting flavour. Their use should be particularly considered because their risk of gelling failure is significantly lower compared to traditional coagulants [68].

Equally important may be the technical role of MBA and CBA as antioxidants. As conventional antioxidants provide not enough protection of powdered oils and fats that are rich in unsaturated fatty acids, Okuno et al. blended them with MBA or CBA to enhance lipid oxidation stability [69].

In the future, both sugar acids may be used in food preservation too. For example, Hu et al. proposed a process to coat fruits and vegetables with a supramolecular film which is formed by non-covalent bonding of small, biomass-derived molecules including MBA [66].

The presented functions indicate the versatile usage of MBA and CBA in food and beverages. However, it should be pointed out that most of the found applications are based on pending patents owned by the company Sun-ei Sucrochemical Co. Ltd. [62–65, 67, 68, 70]. To confirm the functions of both aldobionic acids, further independent investigations should be carried out. Like in pharmaceutics, the long-term effects of their regular ingestion have not yet been considered. Even though their health benefits seem to prove the opposite, it is necessary to assess their health safety, especially in the light of their possible importance in plant-based diets.

### Applications in the chemical industry

Although their employment in chemical industry is still under development, the existing applications make use of the versatility of MBA and CBA.

Inoue et al. proposed a mixture of MBA and one of its salts like MBA-Ca as a stabilizer for cyclohexane monoterpenes [71]. Those molecules belong to the class of terpenes which occur in plants as secondary metabolites [72]. They are used commercially in flavour and fragrance industries and their use in pharmaceutics is under investigation [72, 73]. Additionally, MBA is the key component of a novel chiral selector to separate enantiomers in nonaqueous capillary electrophoresis [74]. This technique uses a solution of an electrolyte and at least one organic solvent at a low current [75, 76]. In this specific case, boric acid served as an electrolyte and the organic solvent was replaced by ionic liquids containing MBA and tetramethylammonium hydroxide because MBA has a low solubility in organic solvents. During a performance test, 21 out of 22 different amino alcohol drugs could be purified and 12 substances showed a satisfying resolution, demonstrating the high enantiomeric selectivity of the chiral selector [74].

In the recent years, the production of biofuels and chemicals from lignocellulosic biomass received growing attention coming along with several advantages. For example, they are derived from agricultural waste and do not compete with food stocks [77]. However, the production is not yet very competitive because the cellulases employed in biomass degradation are expensive and have low conversion rates. Lytic polysaccharides monooxygenases (LPMOs) were recently proposed to overcome the disadvantages of cellulases [78, 79]. Degrading lignocellulosic biomass with LPMOs, aldobionic acids are formed as the main product. They became consequently more important and are investigated as carbon sources for producing biofuels and chemicals. CBA, in particular, displays more benefits than other aldobionic acids, with its lower inhibitory effects on cellulases being especially noteworthy [80]. As a consequence, the production costs could be significantly reduced because a smaller amount of expensive cellulases would be necessary for lignocellulosic depolymerisation [81]. CBA was, therefore, investigated more closely as a substrate for biorefinery. It was reported that *Neurospora crassa* (*N. crassa*) and *Escherichia coli* (*E. coli*) naturally metabolize CBA, leading to the proposal of some biofuel production processes using CBA as substrate [81–83]: Desai *et al.* found intracellular isobutanol production from CBA by *E. coli*, achieving 36% of theoretical maximum yield of isobutanol in 48 h resulting in a product concentration of 1.4 g L^−1^ and a productivity of 0.03 g L^−1^ h^−1^ in a two-step process starting with crude hydrolysate as substrate which is first degraded by *N. crassa* to CBA and then used by *E. coli* for isobutanol production [81]. Another example is the production of ethanol by an engineered strain *Klebsiella oxytoca* WT26 in a co-fermentation process of CBA and glycerol*.* Using a lysogeny broth (LB)-medium, the ethanol yield was about 96% within 36 h, which corresponds to an ethanol concentration of 12 g L^−1^ and a productivity of 0.33 g L^−1^ h^−1^ [83]. In an optimized urea medium which was developed to combine fermentation efficiency and low nutrient costs [84], ethanol yield was 91.2% and an ethanol concentration of 11.3 g L^−1^ was produced in the same period of time, which resulted in a productivity of 0.31 g L^−1^ h^−1^ [83].

The mentioned examples demonstrate the successful use of CBA and emphasise its potential as a possible platform chemical in fermentative biofuel production. It is currently underexploited, likely since pure CBA is very expensive. Two-step processes as demonstrated by Desai et al. would be a valuable solution to evade high substrate costs and make the production of the target product more profitable [81]. Hence, it is important to further investigate the bio-production of CBA.

## Chemical production methods

The oxidation of disaccharides to form aldobionic acids or, more precisely, lactose to LBA was reported by Fischer and Meyer in 1889 using bromine water [85] and the research in this field of chemical methods is still ongoing. Catalytic procedures can be less laborious and complex than biological systems as no sterilisation is necessary and product solutions are simpler to work up. Nevertheless, it must be screened so that by-product formation and their accumulation can be estimated. Additionally, the re-usability needs to be considered as catalysts normally contain noble metals, and thereby are very cost intensive in plants compared to bioprocesses.

This review casts a glance on several methods like electrochemical, microwave-assisted, and heterogeneous catalytic oxidation. A cost rating or economic analysis of the catalysts is necessary to compare those with biological systems, but it would exceed the frame of the review.

As aforementioned, aldobionic acids can be produced by adding bromine as catalyst to an aqueous solution [85–87]. According to a pending patent [86], the bromine was removed with gaseous nitrogen after the oxidation and the solution was neutralised, desalted and then freeze-dried afterwards. The product had a purity of 79.3% [86]. But with the use of this hazardous halogen as catalyst, the production route must be questioned and its use in the pharmaceutical, cosmetics or food sector seems foreclosed.

Alternatively, MBA and CBA can be produced electrochemically. Parpot et al. used a glass cell with a gold electrode for the oxidation initially containing 9.40 mM substrate [88]. Using a sodium carbonate buffered system compared to a solution with 0.1 M NaOH, the conversion of maltose improved to 79% and the selectivity for MBA rose from 27% to an acceptable value of 92%. The most concentrated by-product was maltose dicarboxylic acid in which 3% of the initial amount of maltose remained, but the batch also contained gluconic, formic, glycolic and glyceric acid [88]. This by-product formation can complicate the downstream processing.

The same buffer system was then used for a side experiment with cellobiose. About 95% of the initial concentration of cellobiose was converted to CBA within 20 h. Further information about the conversion as well as by-product formation is neither shown nor mentioned in the supplementary document [88].

Another approach is described by Omri et al. who performed a microwave-assisted catalysis [89]. A 5% aqueous maltose or cellobiose solution with 2.5 mg AuAl_2_O_3_ and a 30% hydrogen peroxide solution, as oxidizing agent, were mixed and irradiated at 60 °C for 20 min. A conversion of 99% with a selectivity of 95% was obtained with both substrates. The re-usability of the gold catalyst was shown by performing five batches without observing greater losses in activity and only small losses of catalyst. What needs to be criticized in this approach: Formation of any side products was not analyzed and no quantification of those was performed in this experiment [89].

Even though the production parameters are impressive for such a short experiment, the possibilities for a scale-up of microwave-assisted productions are limited. It can be assumed that no industrial process would be based on this method.

The last approach discussed here is a catalytic oxidation in an oxygen-flushed reactor. Tomar et al. performed the oxidation using a gold-hydrotalcite-catalyst [90]. The reaction using 71 mM CBA for 3 h at 40 °C was observed. The conversion and by-products are not mentioned as this experiment was meant to screen for other effective conversions with this catalyst. The yield of the experiment was 92.3% [90]. Similarly, Mirescu et al. tested four different catalysts. The initial 10 mM of each aldose were converted by heterogenous catalysis at 40 °C [91]. The Au/TiO_2_ showed the highest activity with 54 and 50 mmol min^−1^ g^−1^ for MBA and CBA. Also in both cases, the selectivity was above 99.5%. The best reaction conditions observed were at pH 9 with a substrate concentration of 200 mM MBA. A recycling test showed a slight decrease in activity from 250 to 150 mmol MBA min^−1^ g^−1^ in over ten batches [91]. Mirescu et al. even claim that no reduction of the catalyst’s activity was observed in 17 glucose-oxidating experiments [88]. Compared to all other reviewed publications, this group’s work seems to be the most promising as the optimised production conditions were identified and re-usability of the catalyst was also proven.

An overview of the reported catalytic processes for the production of MBA or CBA is shown in Table 2.

**Table 2:** Overview of catalytic approaches for the production of MBA or CBA

Catalyst	Catalyst [g L^−1^]	Substrate [mM]	Temperature [K]	Conversion [%]	Selectivity [%]	Volume [mL]	References
**Production of MBA**							
AuTiO_2_	1	10	313	100	> 99.5	600	[91]
PdAl_2_O_3_	1	10	313	100	96	600	[91]
PtAl_2_O_3_	1	10	313	100	91	600	[91]
AuAl_2_O_3_	1	100	313	100	> 99.5	600	[91]
Au	–	10	298	79	92	–	[88]
AuAl_2_O_3_	0.5	146	333	> 99	> 95	5	[89]
**Production of CBA**							
AuTiO_2_	1	10	313	100	> 99.5	600	[91]
PdAl_2_O_3_	1	10	313	100	99	600	[91]
PtAl_2_O_3_	1	10	313	100	87	600	[91]
Au/HT	7.1	71	313	–	92.3*	7	[90]
Au	–	10	298	–	> 95	–	[88]
AuAl_2_O_3_	0.5	146	333	> 99	95	5	[89]

A selectivity marked with a star (*) indicates the yield

Values marked with (–) are neither published nor could be calculated from the published data

## Biological production methods

As already mentioned before, within the biological methods, both whole-cell biocatalysis and cell-free approach have their advantages and disadvantages. This section provides a summary of both types of biocatalysis to produce MBA and CBA.

### Maltobionic acid production

Compared to chemical methods, biological production of aldobionic acids were discovered in the middle of the twentieth century, when Stodola and Lockwood reported their possible biological production [92]. Previously, Lembke had tested the oxidizing action of *Pseudomonas* on reducing disaccharides [93]. Based on this research, Stodola and Lockwood proposed this reaction as a biochemical method to produce aldobionic acids. Plenty of researchers later investigated different types of microorganisms with the same ability, among them are *Pseudomonas* (*P. graveolens, P. fragi, P. quercito-pyrogallica…*)*, Corynebacterium, Zymomonas* (*Zymomonas mobilis*)*, Fungi* (*Acremonium strictum*, *Agaricus meleagris…*) and even plants such as *Iridophycus flaccidum*. The following section includes the most relevant ones up to date.

As already mentioned, Stodola and Lockwood investigated eighteen species of *Pseudomonas* concluding that *P. graveolens* (currently known as *P. taetrolens*)*,* emerging in musty eggs, would act as the best microorganism to metabolize maltose without prior hydrolysis. MBA was isolated as its calcium salts obtaining in 50 h a yield of 77% and 91% purity of the crude fermentation product. A product concentration of 2.5 g L^−1^ could be achieved, which corresponded to a volumetric productivity of 0.05 g L^−1^ h^−1^. *P. fragi* could also oxidize maltose quickly, with only 0.2% of the substrate remaining after 94 h. However, high Ca^2+^ concentrations were measured in the medium suggesting that Ca^2+^ was released and an oxidation of the aldobionic acid took place [89].

*P. graveolens* and *P. fragi* appeared again as ideal whole-cell biocatalysts for high yield production of MBA in the patents of Miyake et al. [94, 95]. In these inventions, whole-cell biocatalysts containing disaccharide dehydrogenase were in charge of the non-growth associated oxidation of highly concentrated solutions which contained only disaccharides or mixtures of mono-, di- and trisaccharides. They found that it was not necessary to add nutrients or nitrogen sources for cell growth and that the reaction can be carried out by simple aeration with air or oxygen. This was a great improvement over prior processes as it avoids the need for a hydrogen acceptor without eliminating its function. Furthermore, it does not require the addition of media compounds that must be removed later as in the fermentation processes. Within these patents, it was possible to achieve a large improvement in the yield, reaching 95–100% of the theoretical yield due to the fact that no by-products such as ketogluconic acid or pantoses, are formed as in the case of fermentation methods using sugar containing media. Nevertheless, a disadvantage is the necessity of separating the cell material from the growth medium before starting the production phase in another medium. This is because during the growth phase, the microorganisms use the maltose as a carbon and energy source, by impeding its conversion [94, 95].

**Fig. 3:**
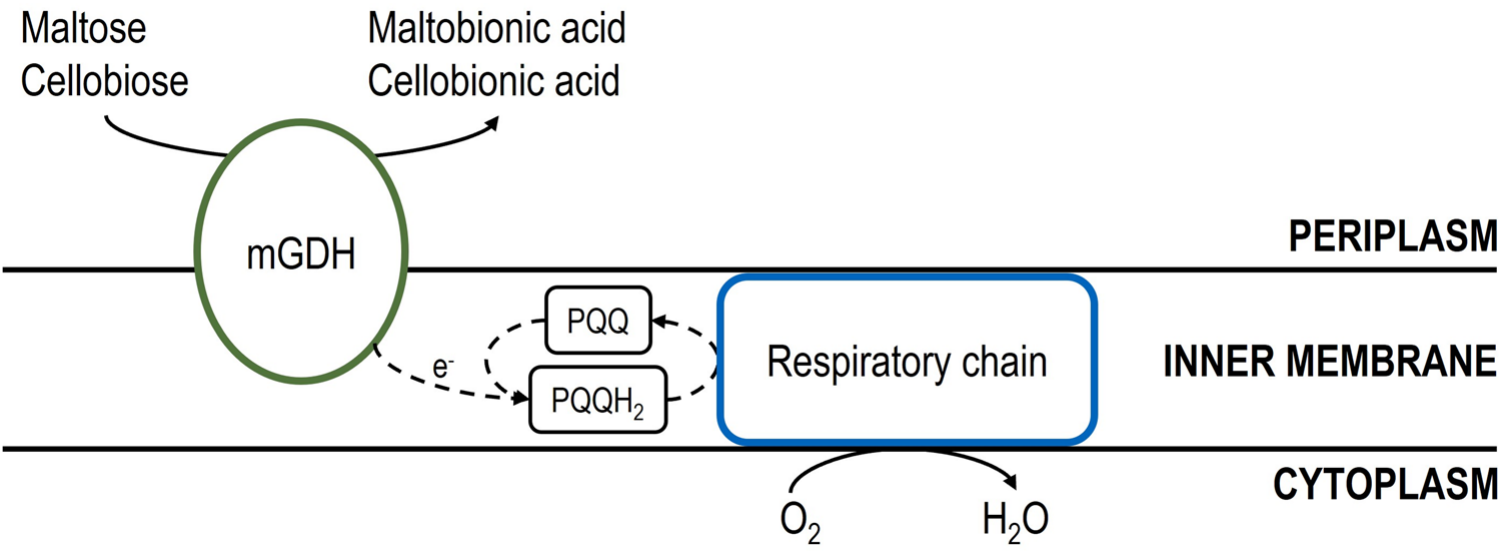
Schematic representation of the reaction mechanism of the membrane-bound glucose dehydrogenase (mGDH), which is responsible for the formation of MBA or CBA in *Pseudomonas* species and acetic acid bacteria [1, 3, 95–97, 101–105]. The pyrroloquinoline-quinone-dependent (PQQ) dehydrogenase is catalyzing the oxidation of maltose or cellobiose. In parallel, the cofactor PQQ is reduced to pyrroloquinoline-quinol (PQQH_2_). The released electrons are transferred into the respiratory chain and are finally transferred to oxygen as terminal electron acceptor, which leads to the formation of H_2_O (derived from [98, 100, 106, 107]). The dashed lines indicate the path of the transferred electrons, while the solid lines represent chemical reactions

Most recently *P. graveolens* has been under study by Oh et al. [1, 96, 97]. They have confirmed that its quinoprotein glucose dehydrogenase (GDH) could convert maltose into MBA after being heterologously expressed in MBA non-producing *E. coli*. They also have expressed GDH homologously in *P. taetrolens* to improve the intracellular maltose-oxidizing activity and, therefore, MBA production. The first results show a 200 g L^−1^ MBA production, 100% yield, and a productivity of 9.52 g L^−1^ h^−1^ in batch fermentation using pure maltose [1]. With high-maltose corn syrup (HMSC), MBA product concentration and yield remains the same, but the productivity decreases to 6.67 g L^−1^ h^−1^. Further investigations and the application of a non-growth associated whole-cell biocatalysis have achieved better productivity with 18.18 g L^−1^ h^−1^ and 8.33 g L^−1^ h^−1^, for pure maltose and HMSC, respectively. This last one was chosen at the end as the better substrate due to the high costs of pure maltose [97]. Moreover, and still under study, the use of waste cooked rice (WCR) as substrate is being explored to revalorize this waste into high value-added MBA [96]. The membrane-bound respiratory metabolism of another *Pseudomonas* species was investigated according to aerobic and anaerobic growth [98, 99]. The respiratory chain of acetic acid bacteria like *Gluconobacter oxydans* is described regarding its genome sequence [100]. Figure 3 should give a general overview of the basic reaction mechanism of a membrane-bound glucose dehydrogenase regarding the formation of MBA and CBA including cofactor regeneration in different bacteria.

*P. fragi* TCCC11892 has been also selected to semi-continuously produce MBA, achieving a product concentration of over 90 g L^−1^ for the first seven cycles. A volumetric productivity of ~ 3.8 g L^−1^ h^−1^ was achieved within each of the first seven cycles. This whole-cell biocatalysis was carried out with 10% (w/w) maltose in a 2 L-bioreactor equipped with a microfiltration membrane for cell recycling. In the first cycle, the cells were recycled back into the fermentation by microfiltration membrane unit after 24 h of bioconversion, and the product stream was obtained at the same time [104].

Still within the genus *Pseudomonas*, Kluyver et al. tested more strains finding that *P. punctata* and *P. zingiberis* were positive for acid formation from maltose. Nevertheless, the results obtained with *P. aromatica and P. quercito-pyrogallica* (named by Beijerinck as *P. aromatica* var. *quercito-pyrogallica* [108]*)* were more remarkable [109]. This batch fermentation showed that the ladder aerobe prokaryote, isolated from canal water, was able to convert maltose into MBA with yields varying between 65 and 80%, which resulted in a product concentration of 63.7 g L^−1^ and a volumetric productivity of 0.17 g L^−1^ h^−1^. More specifically, after 3 days only 12% sugar remained and after 6 days it could already not be measured in the medium. However, an investigation of the later stages of the cultures showed that the formed aldobionic acids vanished slowly. Thus, it was very likely that the microorganism could subsequently cleave the disaccharides into the constituent hexoses [109].

A cell-free bacterial enzymatic system for the oxidation of disaccharides to aldobionic acids has been registered by Bentley and Slechta [110]. With this method, the degradation of the disaccharides can be avoided. A particulate dehydrogenase system from *P. quercito-pyrogallica* is able to produce MBA avoiding the formation of H_2_O_2_. This absence has been considered an improvement because H_2_O_2_ denatures and inactivates the enzyme, causing the system to be rendered useless [110].

An interesting method using 5-aminolevulinic acid (5-ALA) has been proposed to increase the yield of the production with *Pseudomonas* [111]. This strain was inoculated into the liquid fermentation with the addition of 5-ALA in the culture medium. As a result, the conversion ratio was improved and the MBA yield after 24 h was increased from 40 to 95% [111].

One patent for the fermentative production of aldobionic acids by means of the catalase-producing, gram-negative bacterium *P. cepacia* (more commonly known as *Burkholderia cepacia)* was also presented [112]. The 2-phase-process (growth phase and production phase) can use not only disaccharides in pure form, but also products rich in the relevant disaccharide, such as maltose syrups or starch hydrolysates which contain relatively more maltose. The oxidation efficiencies were approximately from 70 to 95%, with mole of disaccharide employed calculated on mole of aldobionic acid produced. The oxidizing activity of *B.* *cepacia* has also been studied by Murakami et al. [113]. In comparison with other enzymes from, for example, *Corynebacterium* (illustrated later), the glucose oxidase from *B.* *cepacia* seemed to not require specific hydrogen acceptors, but only oxygen. A yield of 59.8% was achieved in maltose medium with whole-cell cultures. A small amount of MBA was observed after 8 h. It reached a maximum concentration of 4 g L^−1^ at 22 h, but it decreased rapidly and finally disappeared. They also investigated the oxidizing capability of cell-free purified enzyme of the organism, which showed an activity towards maltose, which most likely resulted in the building of MBA.

Kobayashi et al. reported a new bacterium, which has an enzyme able to produce MBA [114]. More specifically, they purified NAD^+^-dependent maltose dehydrogenase from the cell-free extract of an alkalophilic *Corynebacterium* sp. No. 93–1. After cultivation, the primary product from maltose was evaluated as maltono-δ-lactone and it was converted into MBA, so the product did not contain any keto-group [114]. With this same sequence of reactions, Kido et al. found a new membrane-bound dehydrogenase in the cell-free extract of the bacterium *Serratia marcescens* C886-2, which is a facultative anaerobe and opportunistic pathogen bacterium [115].

**Fig. 4:**
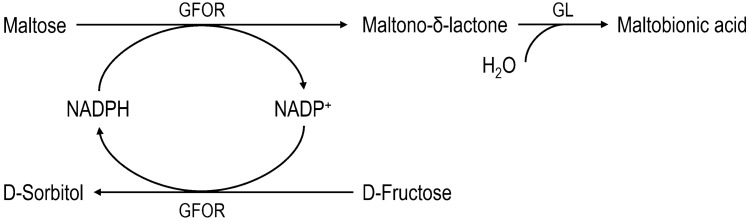
Schematic illustration of the reaction mechanism of the periplasmic GFOR/GL complex of *Z. mobilis*, which natively uses glucose and fructose as co-substrates. Replacing glucose with maltose leads to the formation of MBA, and D-sorbitol, correspondingly. The oxidation of maltose is catalyzed by the enzyme GFOR under reducing its cofactor NADPH to NADP, resulting in the formation of maltono-δ-lactone, which is then converted by the second enzyme GL to maltobionic acid. In a second reaction D-fructose is converted to D-sorbitol via GFOR by parallel regenerating the cofactor (adapted from [121])

Until recently, the genus *Zymomonas* had been barely studied for MBA production. Based on the studies of Satory et al. [116] and previous studies [117, 118], Malvessi et al. have evaluated the periplasmic enzymatic complex glucose-fructose oxidoreductase (GFOR)/glucono-*δ*-lactonase (GL) of permeabilized free and immobilized whole cells of *Zymomonas mobilis* (*Z. mobilis)*. The activity of the GFOR/GL complex within a non-growth associated whole-cell biocatalysis resulted in the bioconversion of mixtures of fructose and maltose into MBA (Fig. 4), achieving 88% conversion of maltose within 24 h, a MBA concentration above 200 g L^−1^ and a volumetric productivity of 8.5 g L^−1^ h^−1^ [119]. Because of the high-value substrate, an alternative maltose source, such as high maltose syrup, was explored later. Immobilizing *Z. mobilis* cells in a matrix of polyurethane resulted in high product concentrations of up to 230 g L^−1^ of MBA with a yield of nearly 90% and a volumetric productivity of 6.75 g L^−1^ h^−1^ using the GFOR/GL complex [120]. The underlying catalytic mechanism of this enzyme complex was first described in 1986 for the conversion of its native substrates glucose and fructose to gluconic acid and sorbitol [121].

Representatives of the eukaryotic domain have been equally found as aldobionic acid producers. Bean and Hassid have reported the first photosynthetic organism with an enzyme, more specific a carbohydrate oxidase, capable of oxidizing maltose to its corresponding aldobionic acid [122]. With cell-free extracts of the red seaweed *Iridophycus flaccidum* and at an optimum pH 5, the formation of MBA can be achieved, consuming O_2_ and forming H_2_O_2_ at the same time.

A glucooligosaccharide oxidase from the fungal mutant strain *Acremonium strictum* T1 was suggested in the 1990s to be immobilized for the continuous enzymatic production of aldobionics acids, being MBA one of them [123–126]. The enzyme, later studied in detail [127], was covalently immobilized to chitosan with polyethyleneimine and glutaraldehyde, improving thermal stability. Under the conversion of starch hydrolysate to aldobionic acids, the enzyme maintained 75% of its original activity after 60 days of continuous operation. It was also found that the immobilized enzyme was less inhibited by high concentration of maltose as substrate [125].

The basidiomycete fungus *Agaricus meleagris* generates pyranose dehydrogenase (PDH), which is described as a quinone-dependent monomeric extracellular flavoglycoprotein [128]. An interesting finding is that this fungal enzyme is capable of catalyzing the substrate-dependent C-1, C-2, C-3, C-1,3′ or C-2,3(′) (di)oxidation of a number of mono- and disaccharides with 1,4-benzoquinone as an electron acceptor. That is why although the oxidation of maltose to MBA takes place, the major final oxidation product with this enzyme is 2,3′-didehydromaltose.

**Fig. 5:**
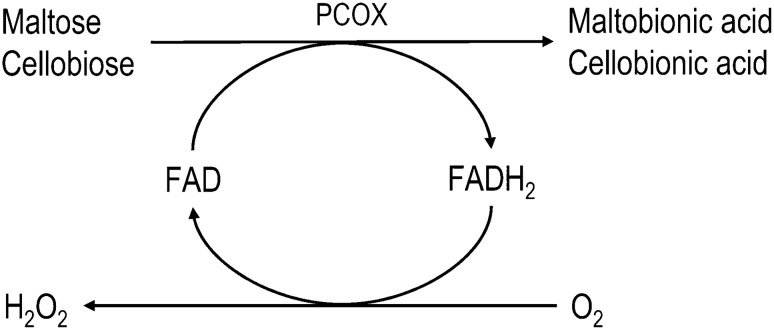
Schematic representation of the reaction mechanism of the secreted carbohydrate:acceptor oxidoreductase from *Paraconiothyrium* sp. (PCOX), which is capable of oxidizing α- or β-1,4-linked disaccharides like maltose or cellobiose. Parallel, the cofactor flavin adenine dinucleotide (FAD) is converted into its reduced form FADH_2_. By transferring electrons to molecular oxygen as terminal electron acceptor, the cofactor FAD is regenerated and additionally H_2_O_2_ is released [130]

A purified carbohydrate:acceptor oxidoreductase from *Microdochium nivale* showed high relative oxidative activities towards maltose and cellobiose [129]. The reaction mechanism of the enzyme is shown in Fig. 5. The by-product H_2_O_2_ should be converted in-situ before causing the deactivation of the enzymes. The so-called *Paraconiothyrium* fungi imperfecti was also investigated for the oxidation of α- and β-1,4-linked sugars studied by Kiryu et al. [130, 131]. They did not investigate deeply the production of MBA or CBA, but for one of their research projects [131], they produced the already mentioned aldobionic acids. For that, they followed the results obtained in a previous study [130]. In that experiments, the ongoing reactions were catalyzed by a carbohydrate:acceptor oxidoreductase from the *Paraconiothyrium*, which belongs to the same group as glucooligosaccharide oxidases (Fig. 5). To determine the substrate specificity, kinetics for various oligosaccharides were measured. The catalytic efficiencies of this enzyme with relevant disaccharides were identified in the following order: cellobiose > lactose > maltose.

In accordance with the previously mentioned patents [95, 112] a new invention presented by Nielsen [132] for the production of MBA has been tested. As the substrate is the starch component of food or feed product, the bionic acid is produced by a two-enzyme catalyzed reaction. The first reaction forms maltose from the starch present during the food or feed production, using an amylase enzyme. For the second reaction, the fungus *Microdochium nivale* is preferred to serve as the producer of the required carbohydrate oxidase, being responsible for the conversion from maltose to the corresponding aldobionic acid. Moreover, catalase is also added to prevent limitation of the reaction driven by the carbohydrate oxidase and to eliminate unwanted H_2_O_2_ in the end-product [132].

### Cellobionic acid production

CBA production has been studied through the cultivation of a wide range of microorganisms, such as *Neurospora crassa*, *Saccharomyces cerevisiae* (*S. cerevisiae*)*, Xanthomonas campestris* (*X.* *campestris*)*, Trichoderma reesei* (*T.* *reesei*)*, Gluconobacter frateurii* (*G.* *frateurii*) and *Peusodomonas taetrolens.* This section summarizes the bio-production methods developed until date.

**Fig. 6:**
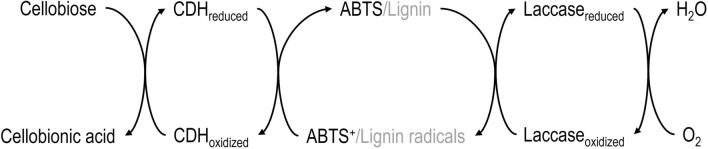
Schematic illustration of the oxidation of cellobiose using the CDH-ATBS-laccase combination for in-situ cofactor regeneration. Cellobiose dehydrogenase (CDH) gets reduced during the oxidation of cellobiose. By applying 2,2-azinobis 3-ethylbenzthiazoline-6-sulfonic acid (ABTS) or lignin from lignocellulosic substrates like wheat straw as redox mediator CDH is re-oxidized. The enzyme laccase is applied to regenerate the redox mediator. Transferring electrons to oxygen, laccase gets re-oxidized (derived from [134, 137, 138])

*N. crassa*, a fungus typically growing in burnt grasslands, is well-known for being an excellent plant cell wall degrader [133]. This microorganism is able to produce a wide variety of cellulases and hemicellulases, but also cellobiose dehydrogenase (CDH) under cellulolytic conditions. This last product is, in turn, capable to oxidize cellobiose to its aldobionic acid, CBA [134, 135]. The oxidation of cellobiose (CB) via CDH requires the presence of a cofactor regeneration system. Hildebrand et al. added the enzyme laccase and a redox mediator to a fermentation using an engineered *N. crassa* strain to produce cellobionic acid from cellulose [134]. As redox mediator they used 2,2-azinobis 3-ethylbenzthiazoline-6-sulfonic acid (ABTS). An optimized recombinant *N. crassa* strain is able to produce cellobionic acid from cellulose without any further addition of enzymes to regenerate cofactors [136]. Additionally, when using lignocellulosic substrate like wheat straw it is possible to produce CBA with an engineered *N. crassa* strain without adding redox mediators like ABTS with a high yield. Zhou et al. found that lignin and lignin degradation products serve as redox mediators [137]. Summarizing, the achieved concentrations of CBA within the fermentation of *N. crassa* ranged from 3.6 to 17 g L^−1^ with corresponding volumetric productivities from 0.02 to 0.43 g L^−1^ h^−1^ [4, 134, 136, 137]. The working principle of the underlying CDH-laccase mechanism including cofactor regeneration is presented in Fig. 6.

Li et al. discovered a novel CBA utilization pathway in *N. crassa*. CBA was identified to be released from cellulose via LPMOs and by CDHs and later, hydrolysed by the secreted β-glucosidase (NCU08755) to glucose and gluconic acid [82]. Authors found that only one of the four major secreted β-glucosidases, NCU08755, showed this activity. However, the deletion of this β-glucosidase decreased CBA consumption. This would imply the existence of a parallel intracellular depolymerization pathway, which means that this filamentous fungus is able to consume both CBA and gluconic acid and that transporters for each must be present. The gluconic acid transporter remains unidentified, but NCU05853 (also referred as CBT-1) was proved to be the transporter for CBA. *S. cerevisiae* has also been investigated for CBA production. This microorganism is one of the few yeasts capable of anaerobic growth even at low pH [139]. Consequently, *S. cerevisiae* does not suffer from phage and other bacterial contamination problems to the same extent as other microorganisms such as *E. coli* [140]. For this reason, the reconstitution of the aldobionic acid utilization pathway in *N. crassa* into *S. cerevisiae* achieved by Li et al. may be economically beneficial [82].

Nihira et al. characterized a novel CBA phosphorylase (XCC4077) from both the cellulolytic bacterium *X. campestris* and the fungus *N. crassa* [141]. They elucidate that this enzyme could perform a critical role in the cellulose degradation, where CBA is converted into α-D-glucose-1-phosphate and D-gluconic acid to enter glycolysis, and the pentose phosphate pathway, respectively.

It has also been suggested that the use of *T. reesei* instead of *N. crassa* improves the yield of cellobiose and cellobionate from cellulose toward fermentable products [4]. *T. reesei*, a mesophilic filamentous ascomycete fungus, is one of the most studied microorganisms for the production of cellulases [142]. It possesses a strong capacity to produce large amounts of lignocellulolytic enzymes [143]. Even though industrial strains and processes have been described to reach enzyme concentrations in excess of 100 g L^−1^, the costly induction of high-level cellulase production is hindering its use [142]. The cellulase production in wild type *T.* *reesei* strains is reliant on expensive media components (i.e., pure cellulose, lactose or sophorose), which make the overall cellulosic ethanol process economically unviable. To reduce the total cost of such processes, low-protease-level strains or protease-deficient strains should be developed or isolated to improve cellulase production.

One of the most efficient producers of CBA has been *Gluconobacter frateurii NBRC3285* [101]*.* The CBA concentration achieved with this bacterium in a whole-cell biocatalysis was 0.86 g L^−1^, which corresponded to a yield of 9% and a volumetric productivity of 0.04 g L^−1^ h^−1^.

Oh et al. recently discovered that the quinoprotein glucose dehydrogenase of *P.* *taetrolens* could also act as a cellobiose-oxidizing enzyme [102]. The homologous expression of this enzyme in *P. taetrolens* allowed the authors to achieve the highest CBA product concentration of 200 g L^−1^ in a whole-cell biocatalysis reported to date. Also, a yield of 95.6% and a productivity of 9.52 g L^−1^ h^−1^ achieved by this recombinant strain after batch fermentation have also improved previous studies. Furthermore, culture conditions such as initial cellobiose concentration, cultivation temperature, and cell density of seed culture were also optimized. Similar state variables, such as pH-control mode, seed culture or oxygen supply, were also described as key in LBA bio-production [144]. A non-growth associated whole-cell biocatalysis with recombinant *P. taetrolens* was investigated for production of CBA. Optimized process parameters were identified regarding temperature, cell density and cell harvest time for biotransformation. Final product concentration of 200 g L^−1^ and the product yield of 95.6% remained the same compared to a growth associated production, but the volumetric productivity could be increased up to 18.2 g L^−1^ h^−1^ [3]. The mechanism of the dehydrogenase, which is capable of oxidizing cellobiose, is illustrated in Fig. 3.

As described above, multiple bacterial species have been investigated to produce CBA. However, there is still an urgent need to improve the CBA product concentration. Furthermore, it is required to implement more economical raw materials for cellobiose production, which is a very expensive substrate compared to lactose or maltose. As a solution to tackle the economic feasibility of CBA production, Oh et al. suggested the preparation of cellobiose from plant biomass and/or wastepaper [3]. In this direction, Yoo et al. converted for the first time waste office paper (WOP) into CBA using a two-step biocatalytic process [103]. Initially, they employed cellulases to convert WOP into cellobiose. To optimize this step, they examined several reaction conditions such as the type and amount of cellulases, reaction temperature and the amount of WOP. From 80 g L^−1^ of WOP, they achieved 23 g L^−1^ cellobiose. In the second step, researchers employed *P. taetrolens* expressing quinoprotein glucose dehydrogenase to generate CBA from the prepared cellobiose. From 23 g L^−1^ cellobiose derived from WOP, this strain produced 24.1 g L^−1^ CBA, which corresponds to a product yield of 100% and a volumetric productivity of 0.86 g L^−1^ h^−1^ [103].

In addition to the cost issue, the conversion of cellobiose to CBA is still a limiting step in those attempts to convert it directly from cellulose. The mechanism is not yet fully understood, although it is known that cellobiose can significantly inhibit cellulase. Thus, shedding light into the conversion of cellobiose to CBA is essential for the utilization of cellulose. An overview of the reported processes for the biological production of MBA and CBA is shown in Table 3.

**Table 3:** Summary of microorganisms investigated for the production of MBA and CBA or for the production of potential MBA and CBA forming enzymes

Microorganism	Method	Enzyme	Conversion [%]	Yield [%]	Product [g L^−1^]	Productivity [g L^−1^ h^−1^]	References
MBA production organisms							
**Bacteria**							
*P. taetrolens* (*P. graveolens)*	Whole-cell	Disaccharide dehydrogenase	97*, –, –	77, 95–100	2.5*, –, –	0.05*, –, –	[92, 94, 95]
		Quinoprotein glucose dehydrogenase	–, –, 100*, -	95.6, –, 100*, 95.6	200, –,168, 200	6.67, –, 5.7, 8.33–18.18	[1, 95–97]
*P. fragi*	Whole-cell	Disaccharide dehydrogenase	98*, –, –, –	–, 95–100,90.5*	–, –, –, 94.7	–, –, –, 3.8	[92, 94, 95, 104]
*P. punctata*	Whole-cell	–	–	–	–	–	[109]
*P. zingiberis*	Whole-cell	–	–	–	–	–	[109]
*P. aromatica*	Whole-cell	–	–	–	–	–	[109]
*P. quercito-pyrogallica*	Whole-cell	–	96.7*	65–80	63.7*	0.17*	[109]
	Cell-free, cell extracts	Dehydrogenase	–	–	–	–	[110]
*P. cepacia (Burkholderia cepacia)*	Whole-cell	–	70–95	–	–	–	[112]
	Whole-cell	–	100	38.2*	4	0.18*	[113]
	Cell-free, crude enzyme	Glucose oxidase	–	–	–	–	[113]
*Corynebacterium sp.*	Cell-free, purified enzyme	NAD^+^-dependent maltose dehydrogenase	–	–	–	–	[114]
*S. marcescens*	Cell-free, purified enzyme	Maltose dehydrogenase	–	–	–	–	[115]
*Zymomonas mobilis*	Whole-cell	GFOR/GL	88, 90*	81.4*, 87	204.1*, 231.9*	8.5*, 6.75*	[119, 120]
**Fungi**							
*A. strictum*	Cell-free, purified enzyme	Glucooligosaccharide oxidase	–	–	–	–	[123–127]
*Paraconiothyrium sp.*	Cell-free, purified enzyme	Carbohydrate:acceptor oxidoreductase	–	–	–	–	[130]
*A. meleagris*	Cell-free, purified enzyme	Pyranose dehydrogenase	–	–	–	–	[128]
*M. nivale*	Cell-free, purified enzyme	Carbohydrate oxidase	> 95	–	–	–	[132]
**Plants**							
*I. flaccidum*	cell-free extracts	Carbohydrate oxidase	–	–	–	–	[122]

Microorganism	Method	Enzyme	Conversion [%]	Yield [%]	Product [g L^−1^]	Productivity [g L^−1^ h^−1^]	References
CBA production organisms							
**Fungi**							
*N. crassa*	Whole cell	–	–, 100, –, –	–	3.6*, 10.2*, 17*, -	0.02*, 0.43*, 0.09*, -	[4, 134, 136, 137]
*Paraconiothyrium sp.*	Cell-free, purified enzyme	Carbohydrate:acceptor oxidoreductase	–	–	–	–	[130]
*Microdochium nivale*	Cell-free, purified enzyme	Carbohydrate:acceptor oxidoreductase					[129]
**Bacteria**							
*G. frateurii*	Whole-cell	Quinoprotein glucose dehydrogenase	–	< 9	< 0.86	< 0.04	[101]
*P. taetrolens*	Whole-cell	Quinoprotein glucose dehydrogenase	–	95.6, 95.6, 100	200, 200, 24.1	9.52, 18.2, 0.86	[3, 102, 103]

Values marked with (*) were calculated from the data published

Values marked with (–) are neither published nor could be calculated from the published data

## Downstream processing

As a part of an industrial production, a suitable downstream process must be established. Due to the simple fact that aldobionic acids except LBA are not produced on a larger scale, the techniques are neither mature nor much literature is available. The focus of researchers is on optimising the production process of MBA and CBA so that in many cases the gathered aqueous solutions were directly analyzed. Tomar et al. applied evaporation [90] and Mirescu and Prüße [91] freeze drying without by-product removal to perform further measurements. In the publications of Mao et al. [104] and Moe et al. [145], the solution was concentrated, then an ethanol precipitation was carried out, the solids were dried, and additional two ethanol washing steps were performed for following measurements.

The patent of Maruo et al. [86] described desalting and freeze-drying after the removal of bromine as downstream process, but the product purity of 79.3% was rather poor. In an active patent, purification is proposed via evaporation and ethanol precipitation followed by drying [111]. According to the authors, the obtained acid is of high purity but yields per step are not mentioned.

In comparison, the downstream processing of LBA is very well described and offers several possibilities. Alonso et al. listed ion exchange chromatography, electrodialysis, and crystallisation as advanced methods [2]. Especially the use of simulated moving bed technology is mentioned as one promising approach to gain high recovery of LBA from a fermentation broth. Borges da Silva et al. reached a recovery of over 99.9% of LBA using this technique [146]. Murakami et al. [147] performed also an ethanol precipitation with a centrifugation afterwards. The crude product was then washed with an ethanol–water mixture whereby a purity of over 99.9% was achieved [147]. In another study varying pH set-points and ethanol amounts were investigated during ethanol precipitation for recovering LBA [148]. Kim et al. obtained the highest recovery rates of LBA after microbial fermentation of up to 97.6% by increasing the amount of ethanol at pH 6.5. Sarenkova et al. compared four different approaches with up to 89% recovery in lab scale experiments [149]. According to their results, a single crystallisation step after microfiltration has the highest yield with 89% but the crystals have only a LBA concentration of 90%. By adding evaporation and ethanol precipitation in the procedure before this operation, the yield decreased to 82% and the purity rose to 95%. The colour of those crystals was closest to the lactose and purchased LBA which also indicates the higher purity. In general, MBA and CBA are very similar to LBA so that those methods should lead to comparable recoveries.

## Conclusion and future perspectives

This review presents the emerging aldobionic acids MBA and CBA, their possible fields of application as well as the state of the art of their production methods with an emphasis on bioproduction. Even if both aldobionic acids show a great versatility in terms of application, they are not used industrially yet. Most likely, their production is still underexplored even though some methods have been already proposed more than 60 years ago [85, 92]. Disadvantages like high catalyst costs have certainly repressed a more intense investigation of chemical production.

Regarding bioproduction, those methods have only recently regained interest. Due to insufficient final product concentrations, high substrate costs and small scales the production of MBA and CBA is not yet feasible at industrial scale. Compared to chemical production methods, biological production of value-added chemicals like aldobionic acids offers some advantages. Whereas chemical synthesis often involves the application of high pressures or high temperatures, biological conversions usually are carried out at ambient pressures and lower temperatures. Additionally, the use of harmful and expensive catalysts can be avoided. Summarizing, bioproduction methods of aldobionic acids seem to be more environmentally friendly and production costs may be reduced if high product concentrations can be achieved. Thus, one major future challenge is the increase of the final product concentrations by focussing on whole cell and enzymatic production approaches. Also, it is necessary to acquire cheap (waste) substrates like it was demonstrated by Oh et al. using cooked rice to generate MBA [96].

Additionally, it has already been shown that the production of CBA from cheap substrates like wheat straw or waste paper is possible [103, 137]. According to this, the direct synthesis from cellulose must be established as standard process to reduce the number of unit operations. One approach could also be a two-step process, in which cellulose is first degraded by fungi into cellobiose, which can then be converted to cellobionic acid by a second microorganism. Another example is the direct conversion of cellulose to CBA by *N. crassa*, which has already been investigated [4, 134, 136, 137].

Although biological methods offer many advantages over chemical synthesis, one major drawback is the challenge to efficiently isolate the products from the fermentation broth. Therefore, it is necessary to develop competitive downstream processes to isolate both aldobionic acids. Equally important is the scale-up of the proposed production methods including feasible downstream processing to the industrial scale since the demand of MBA and CBA may rise in the following years through an increase in the consumption of plant-based food. Due to their versatile properties, MBA and CBA show the potential to replace animal-based ingredients in pharmaceutical, cosmetic, and food applications as well as in chemical industry. Additionally, MBA may be health promoting, which has still to be proven for CBA. Future studies should also confirm the health safety of both aldobionic acids. Further, CBA could serve as a platform chemical for high value added biochemicals or biorefinery [81, 83]. Whether or not MBA shows the same potential still has to be investigated.

## Data Availability

Not applicable.

## Abbreviations

4-CPBA: 4-Carboxyphenylboronic acid
5-ALA: 5-Aminolevulinic acid
ABTS: 2,2-Azinobis 3-ethylbenzthiazoline-6-sulfonic acid
BMD: Bone mineral density
Ca^2+^: Calcium
CB: Cellobiose
CBA: Cellobionic acid
CBT-1: Cellobionic acid transporter
CDH: Cellobiose dehydrogenase
FAD: Flavin adenine dinucleotide
FADH_2_: Dihydroflavin adenine dinucleotide
Fe^3+^: Iron
GDH: Quinoprotein glucose dehydrogenase
GFOR: Glucose–fructose oxidoreductase
GL: Glucono-*δ*-lactonase
GLUT1: Glucose transporter 1
HMCS: High-maltose corn syrup
LB: Lysogeny broth
LBA: Lactobionic acid
LPMO: Lytic polysaccharides monooxygenases
MA: Maltose
MBA: Maltobionic acid
MBA-Ca: Maltobionic acid calcium salt
Mg^2+^: Magnesium
mGDH: Membrane-bound glucose dehydrogenase
PCOX: Carbohydrate:acceptor oxidoreductase from *Paraconiothyrium* sp.
PDH: Pyranose dehydrogenase
PQQ: Pyrroloquinoline-quinone
PQQH_2_: Pyrroloquinoline-quinol
ROS: Reactive oxygen species
UV: Ultraviolet
WCR: Waste cooked rice
WHO: World Health Organization
WOP: Waste office paper
